# Identification of microbial signatures linked to oilseed rape yield decline at the landscape scale

**DOI:** 10.1186/s40168-020-00972-0

**Published:** 2021-01-22

**Authors:** Sally Hilton, Emma Picot, Susanne Schreiter, David Bass, Keith Norman, Anna E. Oliver, Jonathan D. Moore, Tim H. Mauchline, Peter R. Mills, Graham R. Teakle, Ian M. Clark, Penny R. Hirsch, Christopher J. van der Gast, Gary D. Bending

**Affiliations:** 1 School of Life Sciences, The University of Warwick, Coventry, CV4 7AL UK; 2 Rothamsted Research, Harpenden, AL5 2JQ UK; 3 Department of Life Sciences, The Natural History Museum, London, SW7 5BD UK; 4 Centre for Environment, Fisheries and Aquaculture Science (Cefas), Weymouth, Dorset DT4 8UB UK; 5 Velcourt Group Ltd., The Veldt House, Much Marcle, Ledbury, Herefordshire HR8 2LJ UK; 6 Centre for Ecology and Hydrology, Wallingford, Oxfordshire OX10 8BB UK; 7 John Innes Centre, Norwich Research Park, Norwich, NR4 7UH UK; 8 Harper Adams University, Newport, TF10 8NB UK; 9 Department of Life Sciences, Manchester Metropolitan University, Manchester, M1 5GD UK

**Keywords:** Oilseed rape, Microbiome, Rhizosphere, Roots, Landscape, Yield decline

## Abstract

**Background:**

The plant microbiome plays a vital role in determining host health and productivity. However, we lack real-world comparative understanding of the factors which shape assembly of its diverse biota, and crucially relationships between microbiota composition and plant health. Here we investigated landscape scale rhizosphere microbial assembly processes in oilseed rape (OSR), the UK’s third most cultivated crop by area and the world's third largest source of vegetable oil, which suffers from yield decline associated with the frequency it is grown in rotations. By including 37 conventional farmers’ fields with varying OSR rotation frequencies, we present an innovative approach to identify microbial signatures characteristic of microbiomes which are beneficial and harmful to the host.

**Results:**

We show that OSR yield decline is linked to rotation frequency in real-world agricultural systems. We demonstrate fundamental differences in the environmental and agronomic drivers of protist, bacterial and fungal communities between root, rhizosphere soil and bulk soil compartments. We further discovered that the assembly of fungi, but neither bacteria nor protists, was influenced by OSR rotation frequency. However, there were individual abundant bacterial OTUs that correlated with either yield or rotation frequency. A variety of fungal and protist pathogens were detected in roots and rhizosphere soil of OSR, and several increased relative abundance in root or rhizosphere compartments as OSR rotation frequency increased. Importantly, the relative abundance of the fungal pathogen *Olpidium brassicae* both increased with short rotations and was significantly associated with low yield. In contrast, the root endophyte *Tetracladium* spp. showed the reverse associations with both rotation frequency and yield to *O. brassicae*, suggesting that they are signatures of a microbiome which benefits the host. We also identified a variety of novel protist and fungal clades which are highly connected within the microbiome and could play a role in determining microbiome composition.

**Conclusions:**

We show that at the landscape scale, OSR crop yield is governed by interplay between complex communities of both pathogens and beneficial biota which is modulated by rotation frequency. Our comprehensive study has identified signatures of dysbiosis within the OSR microbiome, grown in real-world agricultural systems, which could be used in strategies to promote crop yield.

Video abstract

**Supplementary Information:**

The online version contains supplementary material available at 10.1186/s40168-020-00972-0.

## Background

The world’s population is projected to be over 9 billion by 2050 and will require 60% more food [1]. Up to 80% of this requirement could be met by closing the yield gap of agricultural crops, which represents the difference between the actual and achievable yield [1]. Crops may not reach their achievable yield due to a variety of abiotic factors such as climate or crop management as well as biotic factors [2–4]. In most crops, including maize, wheat, soybean, sugarcane and oilseed rape, frequent cropping on the same land is associated with a decline in yield, of typically between 10 and 30%, and this may be a key contributor to the yield gap [4]. A major factor implicated in yield decline is dysbiosis of the rhizosphere microbiome. Within rotations, break crops are used to disrupt the life cycles of both pathogens and deleterious rhizosphere microbiota, reducing the amount of inoculum that can accumulate within soil. Frequent cropping may result in build-up and carry over of pathogen inoculum, and particularly the development of multi-species pathogen complexes, which may result in a shift from a rhizosphere microbiome which benefits the host, to one which is harmful [4].

Various plant, soil and environmental variables interact with agronomic factors to determine assembly of the rhizosphere microbiome and its effects on crop health [2, 3]. While management of the rhizosphere microbiome has great practical significance for improving the sustainability of agricultural systems, we lack a systematic comparative understanding of the relative importance and interactions of the varied factors which shape the rhizosphere microbiota, and its consequences for crop health and yield, under real-world settings [5–8]. Importantly, despite widespread recognition of the importance of establishing causative links between plant health and the rhizosphere microbiome [9], field-based ecological analysis of the rhizosphere microbiome remains descriptive and functional interpretation of microbiome composition is still largely based on profiling specific microbial taxa which have known beneficial or detrimental impacts on plant health and nutrition, such as pathogens and mycorrhizal fungi [10, 11]

Eukaryotes such as fungi, and particularly protists are largely neglected in studies of the plant microbiome [5, 12–14] despite their important contribution to plant health and regulation of the structure and function of microbial communities [15, 16]. Recent evidence suggests strong eukaryote-bacteria interactions within the rhizosphere which may control community stability and confer host resistance to pathogens [17], emphasising the need for holistic analysis of microbiome composition and interaction pathways when considering rhizosphere functions. Furthermore, studies have largely focused on either the root-associated or rhizosphere soil community, with few comparative studies, despite evidence suggesting that drivers of community assembly in these compartments will be different, reflecting the contrasting importance of direct and indirect plant-interaction pathways [16, 18].

Oilseed rape (*Brassica napus*) is the third most cultivated crop in the UK and the world's third largest source of vegetable oil, with 70 million tonnes produced annually worldwide [19, 20]. Field experiments have indicated that oilseed rape (OSR) yield declines proportionally with the frequency it is grown in rotation, and yield losses of up to 25 % have been reported [21–25]. This has been associated with changes to rhizosphere bacterial and fungal community composition, and increased abundance of a number of putative pathogens [21, 26], but the nature of the microbial interactions which underlie a shift from a beneficial to a deleterious microbiome remain elusive.

In the current study, we used a landscape sampling approach to link the relationship between the below-ground OSR microbiome and plant health. OSR root, rhizosphere soil and bulk soil compartments were sampled from 37 commercial farms in the UK, which were chosen to include a range of OSR cropping frequencies within rotations. Comprehensive analysis of metadata across sites was used to identify the specific management practices, climatic variables and soil physico-chemical properties which determined assembly of each microbial kingdom within the compartments. We identified root and rhizosphere specialist microbial taxa, including several novel rhizosphere protist and fungal clades. Lastly, we characterised microbial co-occurrence patterns within the root and rhizosphere compartments and used these to identify microbial taxa which were positively or negatively associated with both OSR rotation frequency and OSR yield, thereby identifying putative microbial signatures of crop health.

## Materials and methods

### Sample collection

**Fig. 1:**
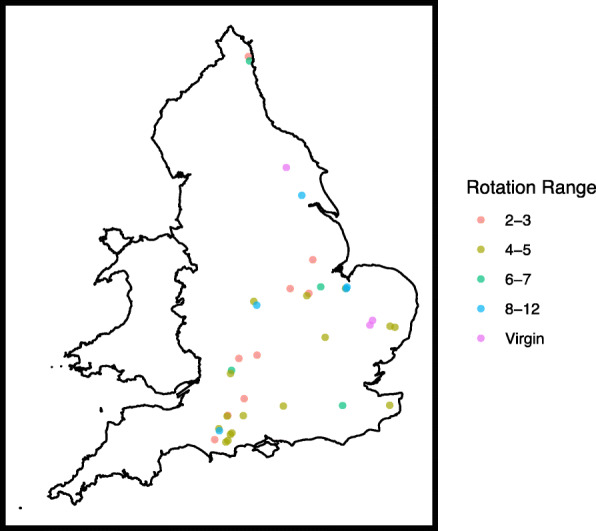
Location of the 37 sites sampled in this study. The rotation range (years since OSR was last grown) is indicated. Virgin is OSR grown for the first time

Thirty-seven OSR field sites from 25 commercial farms located within the main UK OSR growing region were sampled in March 2015 (Fig. 1). Agronomic metadata for each farm was collected including rotation length (years since OSR was grown previously), cropping history, sowing date, variety, pesticide use and the subsequent seed yield (Supplementary Tables 1 and 2). Meteorological data for rainfall and temperature at each field site were obtained from the UK Met-Office (www.metoffice.gov.uk). Soil textural analysis was determined using laser diffraction at NRM Laboratories Ltd.

Samples were taken using a W-shaped sampling pattern starting at least 25 m into the field to avoid edge effects. Five ‘W’ transects were marked out with canes 10 m apart with each ‘W’ measuring 8 m × 4 m. Plants closest to the canes were selected and were removed from the ground with roots attached. Five plants from each ‘W’ were pooled resulting in 5 composite samples per field site. Bulk soil samples were collected from plant-free areas close to the canes using an auger to a depth of 20 cm and were pooled as above. All samples were stored at 4 ^o^C overnight and processed the following day. Loosely adhering soil was removed from the roots leaving no more than 2 mm rhizosphere soil. Approximately 6 g of roots with closely adhering soil were vigorously washed sequentially in 4 × 25 ml sterile distilled water to release the rhizosphere soil which was then centrifuged (3250×*g* for 10 min) and the excess water drained to leave a pellet of rhizosphere soil. Washed roots that were less than 2 mm diameter were cut into approximately 5 mm pieces to yield root samples (comprising of closely associated rhizoplane and endophytes). Bulk soil samples were sieved through a 7 mm, then 2 mm sieve, and approximately 6 g was washed in sterile distilled water using the same sequential washing method as the rhizosphere soil samples to ensure bulk soil and rhizosphere soil samples were comparable.

### Soil chemical analysis

All analysis was carried out using the procedures and methodologies of Rothamsted Research’s Analytical Chemistry Unit. For each replicate, pooled bulk soil samples were sieved through a 2 mm steel mesh and air dried for approximately 2 weeks. Soil pH was measured with an Orion-3-star pH meter (Thermo Scientific). Nitrate, ammonia and available (Olsen) phosphorus concentrations were determined using a Skalar SAN^PLUS^ System, Analytical BV, Breda, Netherlands as per the manufacturer’s instructions [27, 28]. Extractable sulphur was analysed via the Optima 7300 DV Inductively Coupled Plasma - Optical Emission Spectrometer (ICP-OES) (Perkin Elmer Life and Analytical Sciences, 710 Bridgeport Avenue, Shelton, CT 06484 USA) after KH_2_PO_4_ extraction. To determine the concentration of extractable major and trace elements (see Supplementary Table 2) an NH_4_NO_3_ extraction was carried out and analysed by ICP-OES (Perkin Elmer Life and Analytical Sciences, 710 Bridgeport Avenue, Shelton, CT 06484 USA). To detect total major and trace elements (see Supplementary Table 2), approximately 5 g of air dried sieved soil was milled using a Retsch mill PM 400 (Christison Scientific, Albany Road, Gateshead, NE8 3AT, UK) for 6 min at 250 rpm. Aqua regia digestion of soil was performed and the subsequent extract was analysed using an ICP-OES (Perkin Elmer Life and Analytical Sciences, 710 Bridgeport Avenue, Shelton, CT 06484 USA) [29]. A LECO TruMac Combustion Analyser (St. Joseph, MI, USA) was used to measure total N and total C. Inorganic C was determined by phosphoric acid digestion and analysis on a Skalar Primacs Analyser, Skalar Analytical BV, Breda, Netherlands.

### DNA extraction

Root, rhizosphere soil or bulk soil samples (250 mg) were randomised across six 96-well plates and extracted using the PowerSoil-htp™ 96 Well Soil DNA Isolation Kit (MoBio Laboratories, Carlsbad, CA, USA) following the manufacturer’s recommendations, except the samples were homogenised in a TissueLyser II (Qiagen) at 20 Hz for 2 × 10 min with a 180^o^ rotation of the plates between homogenisations. Quality and quantity of DNA was checked on a Nanodrop (Thermo Scientific).

### Sequencing

For each sample, 10 ng of DNA was used to amplify either the fungal ITS2 region (fITS7-ITS4) [30], the V3-V4 region of the bacterial 16S rRNA gene (341F and 806R) [31, 32], or the V1–V3 region of the eukaryotic 18S rRNA gene (Euk-A and Euk-570R) [33]. The primer sets were modified at the 5′ end with adapters from a dual-index sequencing strategy [34]. PCR reactions were performed in a reaction volume of 25 μl, containing Q5® Hot Start High-Fidelity 2X Master Mix (New England Biolabs) and 0.5 μM of each primer. Cycling conditions for 16S were as follows: 95 ^o^C for 2 min, 30 cycles of 95 ^o^C for 30 s, 55 ^o^C for 30 s, 72 ^o^C for 5 min and then final extension of 72 ^o^C for 10 min. Cycling conditions for ITS were as follows: 95 ^o^C for 2 min, 30 cycles of 95 ^o^C for 30 s, 52 ^o^C for 30 s, 72 ^o^C for 2 min and then final extension of 72 ^o^C for 10 min. Cycling conditions for 18S were as follows: 95 ^o^C for 2 min, 30 cycles of 95 ^o^C for 20 s, 57 ^o^C for 15 s, 72 ^o^C for 5 min and then final extension of 72 ^o^C for 10 min. The amplicons were purified and normalised using the SequalPrep™ Normalization Plate Kit (Invitrogen). The libraries were sequenced using the Illumina MiSeq Reagent Kit v3 (600-cycle) on a total of nine MiSeq runs. Following sequencing, Trimmomatic v0.35 was used to remove low-quality bases from the sequence ends [35]. The following steps were then performed using USEARCH and UPARSE software [36, 37]. Paired-ends reads (16S rRNA and ITS) were assembled by aligning the forward and reverse reads and quality filtering (-fastq_maxee 0.5). For 18S rRNA, the forward read alone was truncated to 225 nt due to the large size of the amplicon which meant that reads would not overlap. Unique sequences were sorted by abundance, then singletons (usearch -sortbysize–minsize 2) were discarded from the dataset. Sequences were clustered to OTUs at 97% minimum identity threshold (usearch-cluster_otus), where chimeras are removed using chimaera filters integrated into the algorithm. Further chimeras were removed using -uchime ref and the databases used for taxonomy assignment. Taxonomy was assigned using Quantitative Insights into Microbial Ecology (QIIME 1.8) [38] and the Greengenes reference database (gg_13_8) for 16S rRNA [39], the UNITE database (version 7.0) for ITS [40], or the SILVA database (release 119) (with taxonomies cross referenced with the PR2 (version 4.10.0) database) for 18S rRNA [41, 42]. Bacterial OTUs were retained from the 16S rRNA dataset and OTUs representing mitochondrial and chloroplast 16S rRNA sequences were removed, resulting in 8,633,474 bacterial reads. Fungal OTUs were retained from the ITS dataset resulting in 14,259,717 fungal reads. From the 18S rRNA dataset, sequences from Archaeplastida, fungi and metazoa were removed to leave predominantly single-celled eukaryotes, referred to hereafter as protists, resulting in 4,889,204 reads. To account for differences in sequencing effort, we decided on a random sub-sampling approach, due to the large differences in library sizes (Weiss et al. 2017). The exclusion of reads resulting from this was shown to not change broad patterns as a function of rarefaction depth (Supplementary Table 3). OTU tables were rarefied according to an even sampling depth that resulted in the retention of the majority of samples (over 98%) or at least 1000 reads. This resulted in 5000 bacterial reads, 2000 fungal reads and 1000 protists reads per sample and a total of 14,256 bacterial OTUs, 5714 fungal OTUs and 2150 protist OTUs. Rarefaction curves for the three amplicons are shown in Supplementary Figure 3. More details of the reads and OTUs removed and retained is shown in Supplementary Table 4.

### Statistical analyses

Direct ordination was used to relate the variability in the distribution of microbes to agricultural management practices, soil characteristics, climatic variables (Supplementary Tables 1 and 2) and geographical distance. Principle coordinates of neighbour matrices (PCNM) were used as explanatory spatial variables [43–45] and were calculated from grid coordinates of the sites using GUSTA ME [46]. Community data underwent Hellinger transformations [47] before undergoing direct ordination analyses. Analyses were performed in CANOCO v5.0 [48]. Principal components analysis (PCA) was first used to reduce the number of environmental variables taken forward to further analyses. PCNM and the environmental variables that significantly explained variation in microbial communities were determined with forward selection (999 Monte Carlo permutations; false discovery rate (FDR) *P* < 0.05) and used in redundancy analysis (RDA) [49]. Partial redundancy analysis was performed when both PCNM and environmental variables were significant to summarize the part of species composition variation explained by environmental variables, after removing the effects of geographic distance (PCNM).

Alpha diversity metrics (Fisher’s alpha) and non-metric multidimensional scaling (NMDS) [50] were calculated using the vegan package in R and plots created with ggplot2 [51, 52]. Ternary plots were created with ggtern in R, using the 1000 most abundant OTUs from each taxa [51, 53].

The FUNGuild v1.0 database was used to assign ecological functions (trophic modes) to each OTU [54]. We accepted guild assignments that had a confidence of “highly probable” or “probable” and used the OTUs that assigned to a single trophic mode which resulted in 676 OTUs (11.8% of total OTUs), representing 41.4% of reads. Rotations lengths were binned into groups of short (1 in 2 years to 1 in 4 years), medium (1 in 5 years to 1 in 7 years) and long (1 in 8 years and longer).

Significant differences in Fisher’s alpha and FUNGuild trophic modes were evaluated with the Kruskal–Wallis rank sum test. *P* values were corrected for multiple comparisons with a Dunn’s test using the FDR with the Benjamini–Hochberg method.

### Network analysis

OTUs accounting for the top quartile of relative abundance were filtered to remove those which did not occur in at least 3 of the replicates for each sample type. OTU tables were combined for each compartment, resulting in 4642 OTUs in bulk soil, 4301 in rhizosphere and 2705 in roots which were used in network analysis. Correlations were calculated using the Sparse Correlations for Compositional data algorithm (SparCC), and *P* values calculated using 1000 bootstraps [55]. Networks were produced by retaining edges with a correlation ≥ ± 0.4 and *P* ≤ 0.05 and were analysed in R [56] using the package iGraph [57]. Modules were calculated by the fast greedy algorithm [58]. Within module degree and among module degree were used to assign roles to nodes [59]. Networks were visualised using ggplot2 [51]. Pearson’s correlations between relative abundance of each OTU and rotation length and yield were calculated, and *P* values corrected using FDR. Edges representing a correlation of *P* ≤ 0.05 and *R* ≥ + 0.2 or ≤ − 0.2 to rotation or yield were added. Chord plots were produced in the circlize package in R [56, 60]. Core networks were produced as above using the twenty most abundant OTUs from bacteria, fungi and protists together with yield and rotation length [61].

### Phylogenetic analysis

Phylogenetic trees of novel OTUs were produced. These included the 18S rRNA sequences corresponding to the ITS sequences of novel fungal OTUs as well as the 18S rRNA sequences of novel protist lineages. Further OTUs which were closely related to these (>95% sequence identity) were included, along with their most closely related sequences downloaded from NCBI GenBank. Sequence alignments were generated using MAFFT v.7 (e- ins-i algorithm) [62] and masked to omit ambiguously aligned positions. Phylogenetic analyses were performed on the CIPRES Science Gateway [63]. Maximum likelihood analyses were performed with RaxML v 8 [64, 65].

## Results

### Links between yield and metadata

**Fig. 2:**
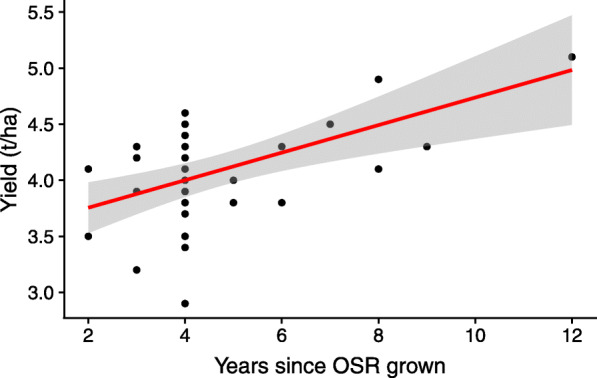
Linear regression showing a statistically significant relationship between seed yield data (collected from each site) and rotation length (years since OSR was last grown) (*P* < 0.001, *R*^2^ of 0.3206). The shaded region is a representation of the 95% confidence limits for the estimated prediction

Of the 53 continuous metadata variables taken (Supplementary Tables 1 and 2), only rotation length (years since last OSR grown) and available potassium (K) showed a significant correlation with yield (FDR *P* < 0.05) (Supplementary Figure 2). Linear regression confirmed a positive relationship between yield and rotation length; *F*(1, 32) = 15.1, *P* < 0.001, *R*^2^ = 0.3206 (Fig. 2).

### Landscape microbial diversity, composition and distribution

**Fig. 3:**
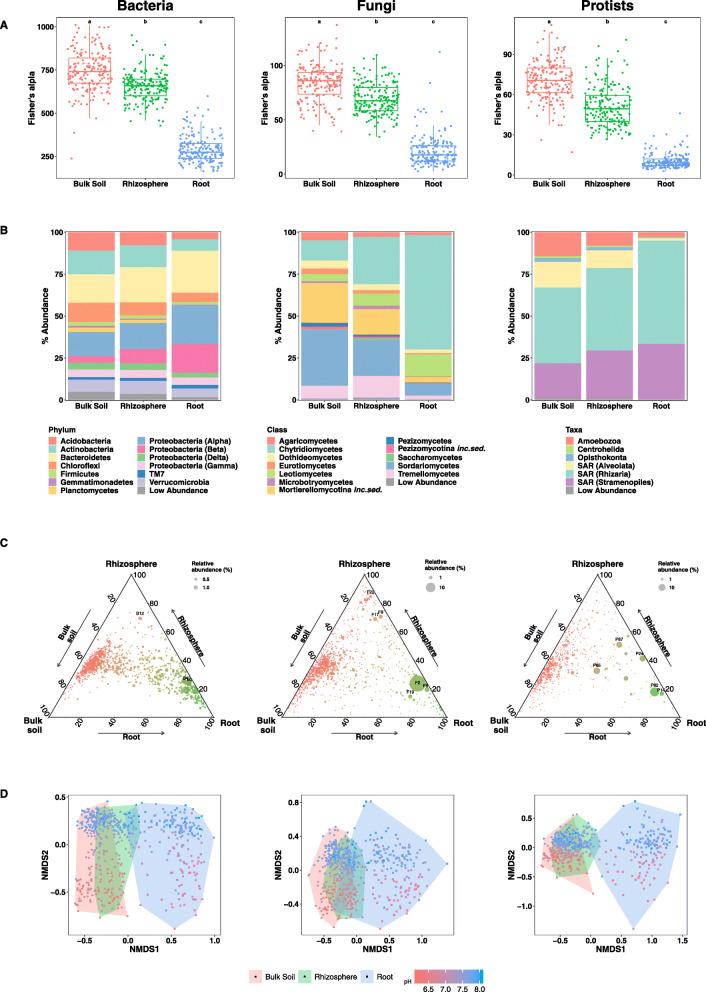
Analyses of bacteria, fungi and protist communities in the three compartments, bulk soil, rhizosphere soil and root. **a** Fisher’s alpha diversity. **b** Stacked barplots showing the relative abundance of taxa. Taxonomic groups with a relative abundance of under 1% in all compartments were combined into the low abundance group. **c** Ternary plots of distribution of OTUs among compartments. B3 = *Methylotenera mobilis*, B4 = *Flavobacterium succinicans*, B12 = *Flavobacterium fluminis*, F2 = *Olpidium brassicae*, F8 = *Cadophora* sp., F9 = *Rozella* sp., F17 = Lobulomycetales sp., F19 = *Tetracladium maxilliforme*, F22 = *Pyrenopeziza brassicae*, P14 = Haliphthorales sp., P24 = *Lagenidium* sp., P65 = *Phytophthora* sp., P82 = *Spongospora subterranean* and P87 = *Spongospora nasturtii*. **d** Non-metric MDS analysis of the microbial community estimated by Bray-Curtis similarity of the bacterial, fungal or protist rRNA amplicons identified to OTU level. Compartments are highlighted by convex hull and point shape. pH of the bulk soil where the sample was taken is shown by the colour scale

In all microbial kingdoms, alpha diversity (Fig. 3a) was significantly greatest in the bulk soil, reduced in the rhizosphere and was lowest in the roots (Kruskal–Wallis, FDR *P* ≤ 0.001). Bacterial diversity was approximately 10-fold higher than that of fungi or protists (Fig. 3a). Taxa which increased in relative abundance as the compartment shifted from bulk soil (BS) through rhizosphere (RH) soil to root (RO) were the bacterial phyla *Proteobacteria* (⍺) (BS = 14.4%, RH = 15.7%, RO = 23.4%), *Proteobacteria* (β) (BS = 4.0%, RH = 8.4%, RO = 17.1%) and *Bacteroidetes* (BS = 16.9%, RH = 21.0%, RO = 25.0%), the fungal classes *Chytridiomycetes* (*Chytridiomycota*) (BS = 12.4%, RH = 24.6%, RO = 64.1%) and *Leotiomycetes* (*Ascomycota*) (BS = 3.5%, RH = 5.9%, RO = 12.5%) and the protist groups *Rhizaria* (BS = 45.1%, RH = 47.2%, RO = 61.7%) and *Stramenopiles* (BS = 20.8%, RH = 28.6%, RO = 33.2%) (Fig. 3b).

For each microbial kingdom, we investigated the distribution of OTUs within compartments using ternary plots (Fig. 3c). The distribution of OTUs was found to vary between microbial kingdoms. All kingdoms possessed a large number of OTUs shared between the bulk soil and rhizosphere soil (red). However, in the bacteria there were also many OTUs predominantly found in the root (green), and a continuum between the bulk soil/rhizosphere and root OTUs (Fig. 3c). There was a distinct lack of a rhizosphere soil selected bacterial community, with only one abundant rhizosphere soil-specific OTU (B12), which had 100% sequence identity to *Flavobacterium fluminis* (*Bacteriodetes*) [KF891387] (Fig. 3c, Supplementary Table 5a).

Within the fungi, dominant root-specific OTUs included F2 which had 100% sequence identity to *Olpidium brassicae* [AB205212], F8 which had 98% sequence identity to *Cadophora* sp. [KT269668] and F19 which had 100% sequence identity to *Tetracladium maxilliforme* [KX610446] (Fig. 3c, Supplementary Table 5b). In contrast to the bacteria, the fungal microbiome had a distinct rhizosphere soil selected community including F22 which had 100% sequence identity to *Pyrenopeziza brassicae* [MF187548], a group of OTUs within the class *Tremellomycetes* and the abundant OTUs F9 and F17 (Fig. 3c). OTUs F9 and F17 had low identity to Genbank sequences, and detailed phylogenetic analysis placed F9 within a clade comprised of members of the genus *Rozella* (*Rozellida*) [66, 67] (Supplementary Figure 3a), while F17 was placed within the newly described order *Lobulomycetales* (phylum *Chytridiomycota*) (Supplementary Figure 3b) [68].

The protists lacked a specific rhizosphere soil selected community but shared several dominant OTUs between the rhizosphere soil and root, which included P87 which had 99% sequence identity to *Spongospora nasturtii* [AF310901] and P24 which had 99% sequence identity to uncultured eukaryotes found in soil and was assigned to the genus *Lagenidium* [LC160286] (Fig. 3c, Supplementary Table 5c). One abundant OTU was found equally in all compartments (P65) and was 98% similar to species within the genus *Phytophthora* [HM161752]. Abundant root-specific protists included P82, which had 98% sequence identity to *Spongospora subterranea* [AY604173] and P14, which had 93% sequence identity to an uncultured Stramenopile extracted from a marine water sample [JQ781890] (Fig. 3c, Supplementary Table 5c). Detailed phylogenetic analysis of P14 placed it and related sequences from this study to a new clade close to the Oomycete orders *Olpidiopsidales* s.l. (which includes parasites of red and brown algae) and *Haliphthorales* (crustacean parasites) (Supplementary Figure 3c).

We used FUNGuild to assign functional roles to OTUs and found a large and highly significant (*P* < 0.001) increase in the relative abundance of pathotroph reads in the root compared with rhizosphere and bulk soil (Supplementary Figure 4). There was also a significantly higher relative abundance of pathotroph reads in the short rotations compared with long rotations in all compartments (*P* < 0.01). Saprotrophs showed the opposite trend regarding rotation length group and had higher abundance in long rotations compared with short rotations in all compartments (*P* < 0.05). There were very few symbiotrophs, which showed no observable pattern (Supplementary Figure 4).

### Drivers of microbial community assembly

We used redundancy analysis (RDA) to relate variability in the distribution of microbiota to explanatory variables (Table 1). Bulk soil pH accounted for most of the community variation in every compartment in all microbial kingdoms, accounting for 14.4–36.8% of the variation in the communities, with differing effects on each compartment for each microbial kingdom (Table 1). The importance of pH in determining community composition across compartment and taxonomic groups was clearly visualised using non-metric multidimensonal scaling (NMDS) of Bray-Curtis similarity (Fig. 3d).

**Table 1:**
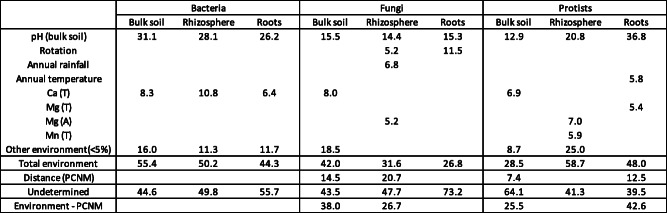
Redundancy analysis determining the percent variation of the bacterial, fungal and protist communities in the different compartments explained by environment and distance (PCNM). Variables which caused over 5% variation in any compartment are included in the table. Nutrients are denoted with either T (total) or A (available). Partial redundancy analysis was performed, when both PCNM and environmental variables were significant, to summarise the part of the species composition variation explained by environmental variables after removing the effects of geographic separation (environment-distance)

Rotation length accounted for 5.2–11.5% of fungal community variation in the rhizosphere soil and roots, but did not account for any variation in bacterial or protist communities. Annual rainfall and annual temperature accounted for a small amount of variation in the composition of the rhizosphere soil fungi (6.8%) and root protist communities (5.8%), respectively. The nutrients Ca, Mg and Mn also contributed to variation across the taxonomic groups (Table 1), with Ca a particularly important contributor to bacterial communities (6.4 to 10.8%) across all 3 compartments. Geographic separation (PCNM) accounted for variation in the fungal bulk soil (14.5%) and rhizosphere soil communities (20.7%), and the protist bulk soil (7.4 %) and root (12.5%) communities (Table 1). Partial RDA (Environment-PCNM), which summarised the part of species composition variation explained by environmental variables after removing the effects of geographic separation, reduced the variation by environment alone, suggesting that the effects of geographic separation were due to a combination of dispersal limitation and environmental differences (Table 1).

### Inter-kingdom co-occurrence networks in OSR microbiomes

To build novel insights into co-occurrence and co-exclusion patterns within the microbiome of OSR at the landscape scale, inter-kingdom microbial interaction networks for the bulk soil, rhizosphere soil and root were generated (Supplementary Figure 5). Connectivity (measured by density) was highest in the bulk soil, lowest in the rhizosphere soil and intermediate in the roots (Table 2). However, modularity was highest in the rhizosphere soil, indicating a greater number of connections within modules than between (Table 2, Supplementary Figure 5). Bacteria formed most of the connections in the multi-kingdom networks, predominantly with other bacteria (Table 2). This was partially due to the larger number of bacterial OTUs inputted into the networks, but accounting for this, a greater proportion of bacterial connections were observed than expected. However, there were a substantial number of inter-kingdom connections, which shifted from predominantly bacteria-fungi to bacteria-protists as the compartment moved from bulk soil, through rhizosphere soil to root (Table 2).

**Table 2:**
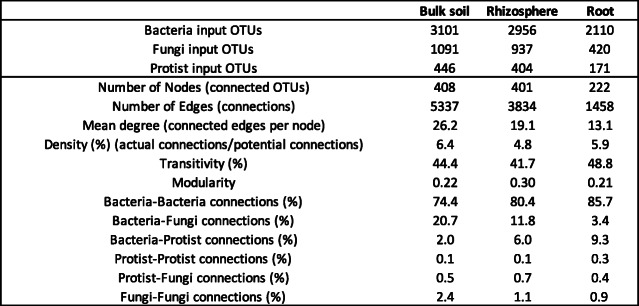
Network statistics for the co-occurrence networks for each compartment. Number of input OTUs used in the network are shown in the top panel

**Fig. 4:**
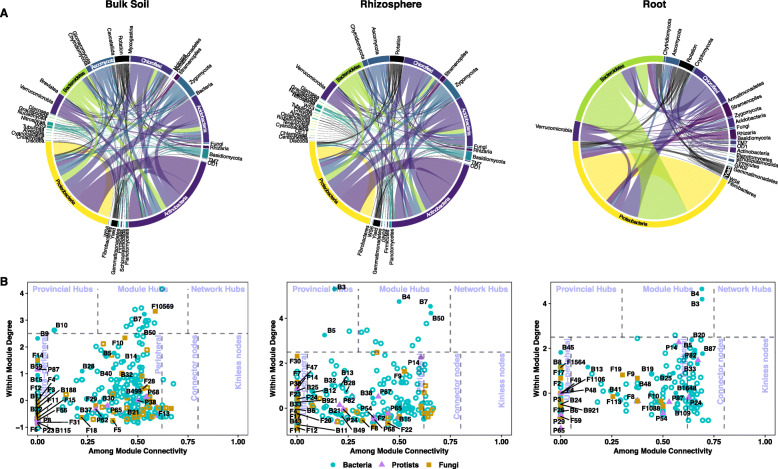
Multi-kingdom OTU networks in the bulk soil, rhizosphere soil and root. **a** Chord plots illustrating the distribution of correlations between high-level taxonomic groups and yield and rotation. The plot includes all correlations, i.e. does not distinguish between positive and negative correlations. Thickness of the ribbon indicates proportion of nodes within each group, which is shown on the outside circle. **b** Network functions of nodes (connected OTUs). Each node was assigned a role according to its topological properties. The within module degree measures how well-connected a node is to other nodes in the same module (*y*-axis). The among module connectivity measures how well-distributed the links of a node are among different modules (*x*-axis). Solid markers indicate the node was one of the 20 most abundant OTUs of their respective kingdom

We visualised the connections between taxa in each compartment using chord plots and included yield and rotation as nodes. These demonstrated that the connections remained remarkably similar between the bulk soil and the rhizosphere soil, which differed substantially to the root (Fig. 4a). In the bulk soil and rhizosphere soil networks, most connections were from the *Proteobacteria* (23%) and *Actinobacteria* (23%), whereas in root networks the *Proteobacteria* formed a larger proportion (45%) of connections, while only 1% of connections involved *Actinobacteria.* There was also greater enrichment of connections involving *Bacteroidetes*, *Chloroflexi*, *Stramenopiles* and *Rhizaria* in the root, relative to the rhizosphere and bulk soil (Fig. 4a). It is noteable that in the roots, the majority of connections associated with yield were with the *Proteobacteria* and *Bacteroidetes*.

Nodes (representing connected OTUs) were assigned network functions based on their among and within module connectivity (Fig. 4b). These allowed us to identify hubs (highly connected nodes), likely to act as keystone taxa that drive and maintain community structure and function. Within the bulk soil networks, hubs included the highly abundant bacterial OTUs B7, B10 and B50, which all had 100% sequence identity to uncultured bacteria isolated from soil and were assigned to the *Cytophagaceae*, *Acidobacteria* and *Skermanella*, respectively (Fig. 4b, Supplementary Table 5). B7 (*Cytophagaceae*) and B50 (*Skermanella*) were also hubs in the rhizosphere soil along with B3, which had 97% sequence identity to *Methylotenera mobilis* [NR_102842] and 100% identity to other uncultured bacteria found in soil, leaf litter and freshwater [69], B4 which had 99% sequence identity to *Flavobacterium succinicans* (*Bacteriodetes*) [MG575969], and B5 which had 100% sequence identity to *Bradyrhizobium* sp. [MH118326]. The only hubs retained in the root network were B3 (*Methylotenera mobilis*) and B4 (*Flavobacterium succinicans*) (Fig. 4b).

Within the fungi, the most connected OTUs (F10, F10569) in the bulk soil were assigned to *Mortierella*, with F10569 designated a module hub (Fig. 4b). In the rhizosphere soil, there were no designated module hubs; however, F9 (*Rozella* sp.) had 64 connections, notably higher than the average of 2 connections for the other highly abundant OTUs (Fig. 4b, Supplementary Table 5b). The most connected fungal OTU in the root compartment was F19 (*Tetracladium maxilliforme*) with 29 connections (Fig. 4b, Supplementary Table 5b).

Several protists had a very high number of connections, although none were designated as hubs. In the rhizosphere soil and root, the most connected was P14 (*Haliphthorales* sp.) (95 and 73 connections respectively). The only protist to have more connections in the root than in rhizosphere soil was P82 (*Spongospora subterranea*) (44 and 17 connections respectively) (Supplementary Table 5c). There were several other highly connected protists in the rhizosphere including P87 (*Spongospora nasturtii*) (45 connections) and P24 (*Lagenidium* sp.) (24 connections) (Supplementary Table 5c, Fig. 4b).

### Correlations with yield and rotation

**Fig. 5:**
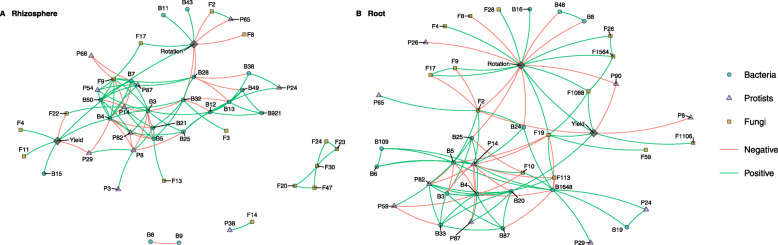
Core networks comprising of the twenty most abundant OTUs from each kingdom including yield and rotation as nodes. **a** Rhizosphere, **b** Root. Pearson correlation coefficients and *P* significance values to yield and rotation are shown in Supplementary Table 5

We produced core networks to explore connections between the most abundant OTUs in the rhizosphere and root (from Supplementary Table 5c) and correlations with yield and rotation (Fig. 5). Rotation length and yield generally did not correlate with network hubs or highly connected nodes in the rhizosphere or root and tended to separate from the main network. However, there were the exceptions of hub B4 (*Flavobacteria succinicans*) which had a positive correlation with yield in the rhizosphere soil, and also highly connected F19 (*Tetracladium maxilliforme*) which had a positive correlation with both rotation length and yield in the root (Fig. 5).

Most correlations with yield and rotation length in the root were with less connected but abundant OTUs (Fig. 5). The relative abundances of several OTUs were correlated with both yield and rotation length, including the fungus *O. brassicae* (F2) and the amoebozoan *Ceratiomyxella tahitiensis* (P90) which increased in relative abundance in shorter rotations, and were associated with reduced yield, while *Tetracladium maxilliforme* (F19) and *Tetracladium furcatum* (F1088), showed the reverse trend, increasing in relative abundance with higher yields and longer rotations. Notably, no bacterial OTUs were associated with both yield and rotation length (Fig. 5).

The relative abundance of a range of OTUs was correlated with rotation length but not yield in root or rhizosphere soil compartments. Bacterial OTUs (B28, B32, B25, B8, B48 and B24) from a range of phyla, together with *Cadophora* sp. (F8), *Lagena radicola* (P26) and *Phytophthora* sp. (P65) had increased relative abundance in shorter rotations (Fig. 5). In contrast, several OTUs increased in relative abundance in the root or rhizosphere soil in longer rotations, including two *Tetracladium* OTUs (F26 and F1564), *Gibbelulopsis nigrescens* (F4), *Rozella* sp. (F9), *Lobulomycetales* (F17), *Mortierella* (F28) and several bacterial OTUs (B11, B43 and B16).

Similarly, a number of OTUs in either the root or rhizosphere compartments correlated with yield but not rotation length. This included *Pyrenopeziza brassicae* (F22), *Glissomonadida* (P8) and *Protaspis grandis* (P29) which increased in relative abundance with reduced yields, while increased yield was correlated with the increased relative abundances of *Flavobacterium* OTUs (B4, B15), *Rhodobacter* (B20), *Gibellulopsis nigrescens* (F4), *Podospora* (F1106) and *Botryotrichum* (F11) (Fig. 5).

Although direct correlations were sparse between highly connected nodes and rotation and/or yield, they were however connected to yield and rotation via other nodes. For instance, the highly connected protist P14 (*Haliphthorales* sp.) had no direct correlations with yield and/or rotation, but correlated via other nodes. These included a negative correlation to F19 (*Tetracladium maxilliforme*) and a positive correlation with F2 (*O. brassicae)* in the root, and a negative correlation with B4 (*Flavobacteria succinicans*) and a positive correlation with F22 (*Pyrenopeziza brassicae*) in the rhizosphere soil (Fig. 5).

## Discussion

Local scale field experiments have previously indicated that OSR yield is affected by rotation frequency [21, 70]; however, it remained unknown whether this phenomenon occurs in real-world cropping systems. Using a landscape sampling approach, incorporating commercial farms across a wide geographical area that use differing soil types and management regimes, we have characterised factors which shape the rhizosphere microbiome of OSR and its relationship with crop health. This comprehensive study revealed that specific environmental and agronomic drivers of community assembly vary between microbial kingdoms and between root and rhizosphere compartments. Importantly, we show that at a landscape scale, OSR yield was associated with rotation frequency, and we have identified key microorganisms, particularly root associated fungi, which are associated with yield and/or rotation, and which could be putative indicators of crop health.

In particular the relative abundance of *O. brassicae* (F2) increased in roots in shorter rotations that suffered from reduced yields, while *Tetracladium maxilliforme* (F19) and *Tetracladium furcatum* (F1088), showed the reverse trend. While correlation does not prove an association with yield, several factors point to these fungi being important determinants of plant health in the field. First and foremost, across all farm sites, *O. brassicae* was the single most abundant component of the root associated microbiome, and has also been found to be the only member of the fungal core microbiome of Canola in a field experiment in Canada [71]. Secondly, our earlier work showed that *O. brassicae* increased in abundance as OSR rotation frequency increased at an experimental field site in the UK, and we further showed that it can reduce OSR growth in glasshouse bioassays [21]. *Tetracladium* spp. are known as aquatic hyphomycetes, but have frequently been detected as endophytes in roots of a variety of crop species [72, 73] and proven to be biologically active in this niche [74]. Here, we show that multiple *Tetracladium* OTUs are not only widespread but also abundant within the roots, but not the rhizosphere soil of OSR. Importantly, the relative abundance of the two most abundant *Tetracladium* OTUs positively correlated with both yield and rotation. While *Brassicaceae* are unable to produce mycorrhizal associations, some *Helotiales* fungal root endophytes related to *Tetracladium* have been shown to act as plant symbionts which promote growth and P supply [75]. Together, our data collected from field-grown OSR at the landscape scale, reveals *Tetracladium* spp. likely provide beneficial functions to the plant affecting its yield.

In addition, a variety of fungal generalist plant pathogens were also found to be abundant and widespread members of the OSR microbiome. This included, *Cadophora* sp. (F8), *Fusarium merismoides* (F5), *Intersonilia* sp. (F79), *Pyrenochaeta* sp. (F59) and *Pyrenopeziza brassicae* (F22). The oomycete plant pathogens *Lagena radicola* (P26) and *Phytophthora* sp. (P65) were also abundant in both roots and rhizosphere soil. Relative abundance of *Cadophora* sp. in both the rhizosphere and root compartments, and *Phytopthora* sp. and *Lagena radicola* in the rhizosphere and roots respectively, increased with short rotations. Enrichment of these pathogens with increased cropping of OSR in the rotation provides evidence that yield decline is associated with the development of a deleterious rhizosphere microbiome comprising multiple pathogens. While the remaining pathogens had no association with OSR rotation frequency, they had relatively low occupancy within the root, suggesting drivers other than rotation frequency were responsible for local abundance. These fungi have the potential to affect yield locally and add to the pathogen pressure which develops by repeated cropping of OSR. For example, *Pyrenochaeta* sp. has been shown to be locally abundant in OSR rhizosphere and roots and have a negative correlation with yield [70], and also has the potential to reduce growth of OSR in laboratory bioassays [21].

Although many OTUs were not assigned a trophic mode during FUNGuild analysis, there was still a significant increase in pathotrophs in the root and in short rotations in each compartment. Saprotrophs showed the reverse trend, and were more abundant in the long rotations. Interestingly *Tetracladium* was classified as a saprotroph rather than a symbiotroph, which could partly account for the increase in saprotrophs detected in long rotations.

While the bacterial and protist communities were not influenced by rotation, there were abundant OTUs which correlated with yield and/or rotation. A single root *Rhodobacter* OTU (B20) and two rhizosphere soil *Flavobacteria* (B4 and B15) positively correlated with yield, but none of these taxa correlated with OSR rotation frequency. While the protists *Glissomonadida* (P8) in the root and *Protaspis grandis* (P26) in the rhizosphere correlated with low yield. Furthermore, an Amoebozoan related to *Ceratiomyxella tahitiensis* (P90) correlated to both increased OSR cropping frequency and reduced yield. The functional significance of these associations is unclear. Pathogenic and mutualistic associations are the best understood rhizosphere interactions, although microbes can affect plant health through other mechanisms such as effects on nutrient availability and microbe-microbe competition. While several *Amoebazoa* are known as human pathogens, most are considered saprophytes [76], and similarly the fungi *Podospora* and *Botryochum* (which both correlated with high yield) are known saprophytes. Decomposition in the root zone by saprophytes could be associated with nutrient availability, providing a feedback loop which could benefit plant health [77]. Interestingly, relative abundance of *Gibellulopsis nigrescens* in rhizosphere soil correlated with high yield, and in roots was linked with long rotations. *G. nigrescens* is considered a saprophyte and under some circumstances a weak pathogen [78, 79], but it has been shown to provide protection against plant infection by virulent *Verticillium* pathogens [79, 80], and similarly our evidence suggests it could represent a beneficial component of the plant microbiome.

Clearly the effect of the microbiome on crop yield reflects the outcome of complex interaction pathways involving mutualistic and antagonistic biota. Microbial network analysis fills a critical gap in our understanding of soil microbial community assemblages by providing insight beyond microbial diversity *per se*, allowing us to visualise co-occurrence and potentially to identify taxa which maintain community structure and function [81, 82]. Bacteria were the only group which formed highly connected hubs in the roots and rhizosphere, and bacteria-bacteria connections dominated interaction pathways, increasing proportionally from the bulk soil, to rhizosphere soil through to the root. This was accompanied in the root with a marked decrease in bacteria-fungal connections and increase in bacteria-protist connections, and relatively high protist connectivity, and low fungal connectivity. Despite the lack of eukaryotic hubs, we have identified a number of highly connected novel fungal and protist OTUs, and evidence suggests that these are abundant, and widely distributed within the microbiome, and therefore may play a role in structuring the microbiome. Of particular note is P14 which is most closely related to the clades *Olpidiopsidales* and *Haliphthorales*, which include parasites of red algae and crustaceans. P14 was by far the most connected protist in both the roots and rhizosphere soil and had a positive relationship with the pathogen *Pyrenopeziza brassicae* in the rhizosphere and the pathogen *O. brassicae* in the roots and a negative relationship with the highly connected and potentially beneficial endophyte *Tetracladium maxilliforme* in the roots, suggesting it could play a role in plant health via indirect interaction pathways. The most connected fungus in rhizosphere soil was a novel clade of *Rozella* (F9), which are considered to be endoparasites of oomycetes and fungi [83], and since F9 had a positive association with yield, there is the intriguing possibility that it represents an endoparasite of the fungal pathogens which were abundant in OSR roots.

Microbes have potential to reduce or enhance each other’s growth via numerous direct and indirect pathways. While practical use of microbial biocontrol agents to control pathogens is well established [84], recognition that microbial pathogens co-occur and can interact synergistically together, and with non-pathogenic microbes to increase disease is only recently being recognized [85]. Using synthetic communities, Duran et al. [17] suggested that the bacterial microbiome provides protection of the plant against fungal and oomycete pathogens via antagonistic interaction pathways, and in particular detected strong competitive potential among a range of taxa including *Flavobacteria* and *Pseudomonads*. However, the pathogens we detected had limited connectivity with other taxa. Despite this, *Flavobacteria* (B4) was highly connected and had a positive correlation with yield in the rhizosphere and a negative correlation to P14 which as mentioned above had a positive relationship with the pathogen *Pyrenopeziza brassicae* in the rhizosphere and the pathogen *O. brassicae,* suggesting these antagonistic relationships may be present. Also, the pathogens *O. brassicae* and *Phytophthora* (P65) were positively correlated in both the rhizosphere and root networks, providing some support for a potential synergism between these pathogens at the landscape scale.

Our study shows that the microbiomes of the three compartments, across the landscape, clustered into well-defined groups, indicating that similar communities were selected into the rhizosphere soil or root at different sampling locations, irrespective of soil and climate variation. Within-compartment community similarity decreased from bulk soil through rhizosphere soil to root, in which there was increasingly greater stochasticity in community composition as complexity declined, with fungi and protists dominated by small numbers of OTUs. This reduction in diversity is likely due to microbial specialization required for invasion and survival inside plant tissue [86]. Notably, there was a distinct fungal rhizosphere soil selected community, which was not the case for bacteria or protists. Fungi which were more abundant in rhizosphere soil relative to roots and bulk soil included the pathogen *Pyrenopeziza brassicae* and novel and abundant OTUs F9 (*Rozella* sp.) and F17 (*Lobulomycetales*), identified here for the first time, demonstrating the importance of sampling the rhizosphere soil and the root as separate compartments.

Significantly, bulk soil pH was the major driver of rhizosphere and root community composition, suggesting that soil pH could override effects of the plant on microbial community assembly. pH has been shown to be a strong predictor of bulk soil bacterial richness, diversity and community composition across landscapes [87, 88], and similarly can be a key factor shaping bulk soil fungal communities [89, 90]. Much less is known about variation of soil protist communities, but evidence also points to pH as a determinant of bulk soil community composition [91]. Furthermore, the microbial groups responded differently to bulk soil pH as a driver of composition within the roots and rhizosphere. In bacteria, the effect of bulk soil pH decreased as the compartment moved from bulk soil through rhizosphere soil to root. In fungi, bulk soil pH was an equally important driver in all compartments. However, in protists, bulk soil pH had the greatest effect on communities inhabiting the root compartment. Importantly, despite pH being a major driver of microbial community composition, there was no correlation between soil pH and crop yield.

## Conclusions

Our data indicates that at the landscape scale, OSR crop yield is governed by interplay between complex communities of both pathogens and beneficial biota which is modulated by rotation frequency. Our work defines a range of potential plant-beneficial and deleterious organisms, including several novel fungal and protist clades which we describe, which could be used to devise strategies to improve plant health. Importantly, this study demonstrates agronomic management, such as crop rotation, plays an important role in promoting beneficial microbes, and reducing pathogens. Targeted isolation of these newly identified beneficial biota, such as strains related to *Tetracladium* spp. and *Flavobacterium* is critical to develop our understanding of plant-microbe and microbe-microbe interaction mechanisms. This will provide a platform to devise novel strategies to promote plant health [92]. These approaches are critical to provide innovative solutions for the sustainable improvement of plant health and crop yield.

## Supplementary Information

**Additional file 1: Supplementary Table 1.** Metadata from the 37 field sites from the 25 farms. Farms with more than one field location are donated with a-d.

**Additional file 2: Supplementary Table 2.** Bulk soil properties from each of the five reps of the 37 field sites. pH, soil water content, bulk density and 35 nutrients are shown. Farms with more than one field location are donated with a-d.

**Additional file 3: Supplementary Table 3.** A comparison of three samples, one bulk soil (BS), one rhizosphere (RH) and one root (RO), rarefied at 1000 reads and not rarefied, to demonstrate the similarity in community composition.

**Additional file 4: Supplementary Table 4.** Sequencing read and OTU statistics for each amplicon (16S, ITS, 18S) and compartment (bulk soil, rhizosphere, root).

**Additional file 5: Supplementary Table 5.** Twenty most abundant OTUs in rhizosphere soil and root compartments, (a) bacteria, (b) fungi, (c) protists. Occupancy shows the percentage of samples containing the OTU. OTU names and identities (%) for (b) fungi and (c) protists were obtained from NCBI blastn top hits.

**Additional file 6: Supplementary Figure 1.** Rarefaction curves of a) Bacteria (16S) at 5000 reads, b) Fungi (ITS) at 2000 reads and c) Protists (18S) at 1000 reads.

**Additional file 7: Supplementary Figure 2.** Correlogram showing significant Spearman correlations (FDR *P* ≤ 0.05) among metadata parameters. Circles are coloured according to the R^2^ value on the sliding scale (blue = positive correlation, red = negative correlation). Suffixes for nutrients, T = total nutrient, A = available nutrient.

**Additional file 8: Supplementary Figure 3.** Phylogenetic tree of 18S rRNA sequences a) including the 18S sequence of the fungi (a) F9 and (b) F17 and the novel protist (c) P14. Other closely related less abundant OTUs found in this study are also included and highlighted in blue. The hosts the lineages are associated, or which environments they were sequenced from are in parenthesis.

**Additional file 9: Supplementary Figure 4.** Relative abundance of trophic modes based on FUNguild determinations. Error bars represent standard error of the mean. Different letters above the bars indicate significant differences at the P < 0.05 level between bars of the same trophic mode. Rotations lengths were binned into groups of short (1 in 2 years to 1 in 4 years), medium (1 in 5 years to 1 in 7 years) and long (1 in 8 years and longer).

**Additional file 10: Supplementary Figure 5.** Correlation networks generated using SparCC. Edges indicate correlation of >0.4 or <-0.4. (a) bulk soil, (b) rhizosphere soil, (c) root.

## Data Availability

The datasets generated and/or analysed during the current study are available in the NCBI Sequence Read Archive under BioProject ID PRJNA548438. The sequences of OTUs highlighted in this study are deposited in the NCBI GenBank database under accession numbers MN045353-MN045395 (16S rRNA), MN047171-MN047210 (ITS) and MN046118-MN046154 (18S rRNA).
