# Supplementary figures and images for: Identification of microbial signatures linked to oilseed rape yield decline at the landscape scale
Source: Microbiome. 2021 Jan 22;9:19. doi: 10.1186/s40168-020-00972-0 (PMC7825223; doi:10.1186/s40168-020-00972-0)

Fig S1.

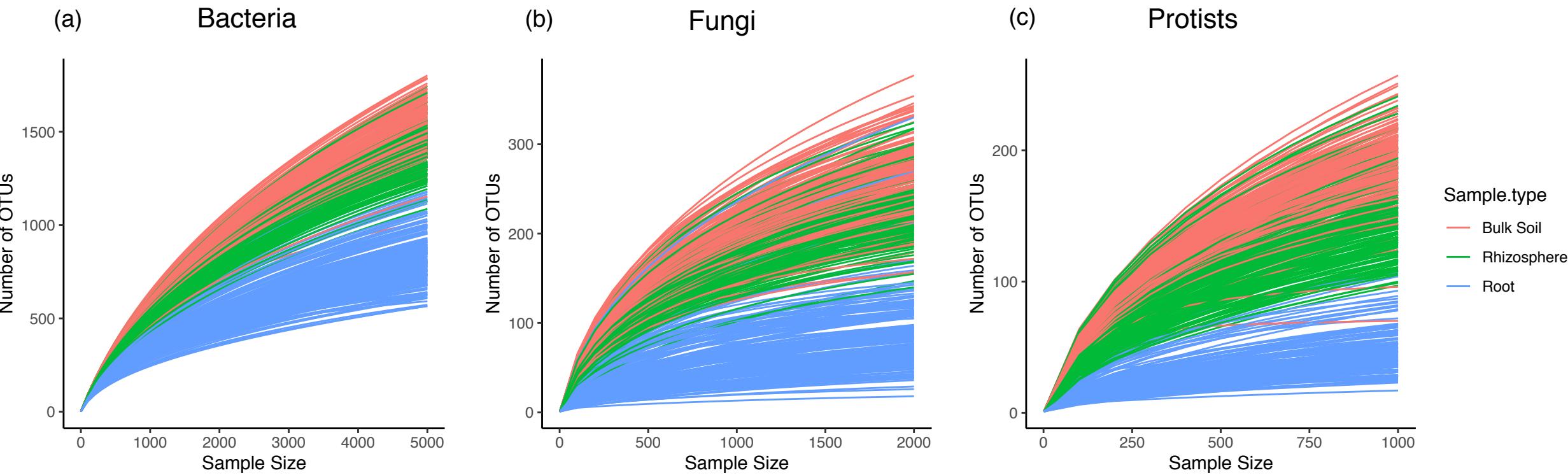

Supplement: Supplementary file 7 — Additional file 6: Supplementary Figure 1. Rarefaction curves of a) Bacteria (16S) at 5000 reads, b) Fungi (ITS) at 2000 reads and c) Protists (18S) at 1000 reads. [file 40168_2020_972_MOESM7_ESM.pdf]

Fig. S2

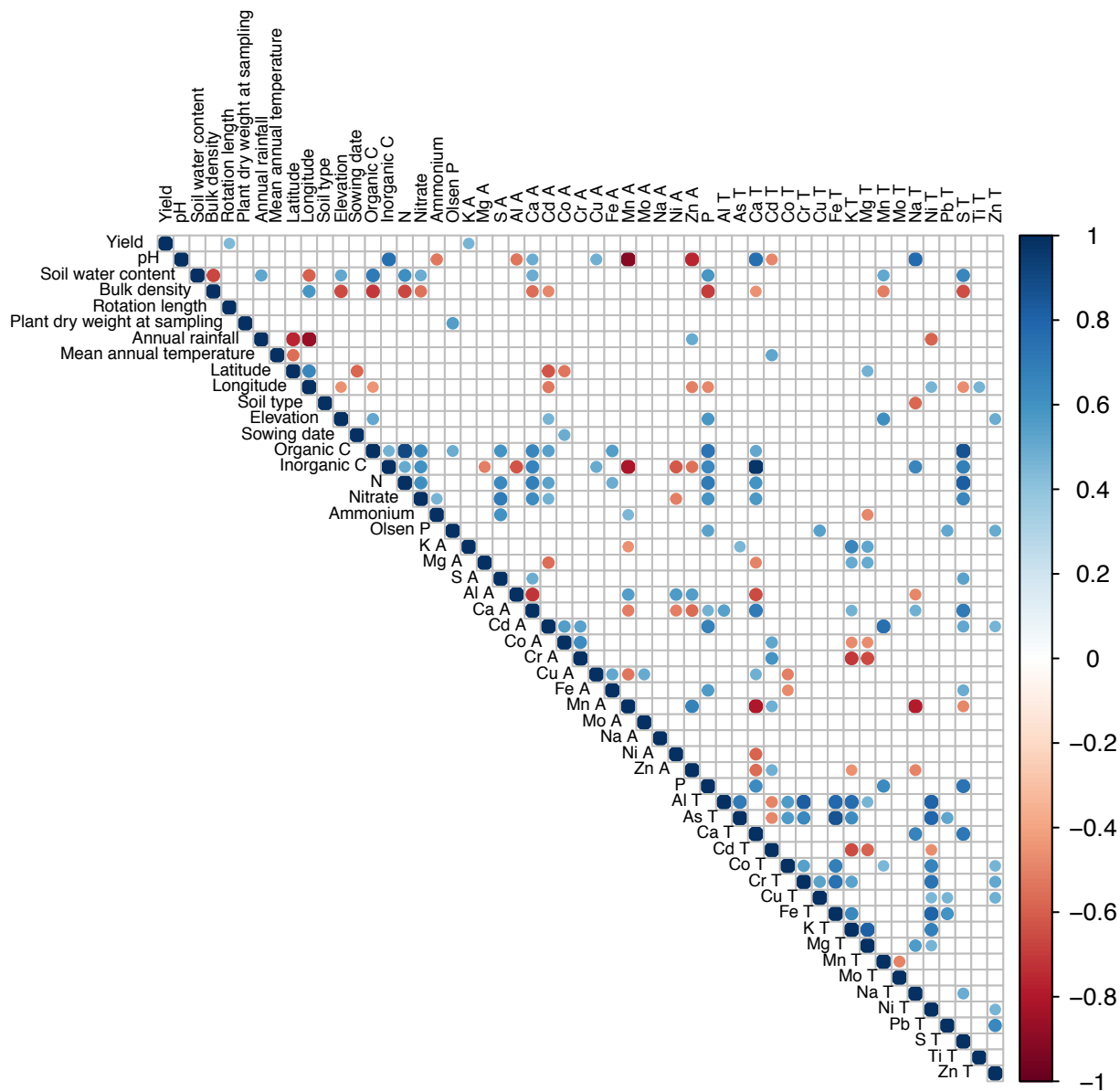

Supplement: Supplementary file 8 — Additional file 7: Supplementary Figure 2. Correlogram showing significant Spearman correlations (FDR P ≤ 0.05) among metadata parameters. Circles are coloured according to the R2 value on the sliding scale (blue = positive correlation, red = negative correlation). Suffixes for nutrients, T = total nutrient, A = available nutrient. [file 40168_2020_972_MOESM8_ESM.pdf]

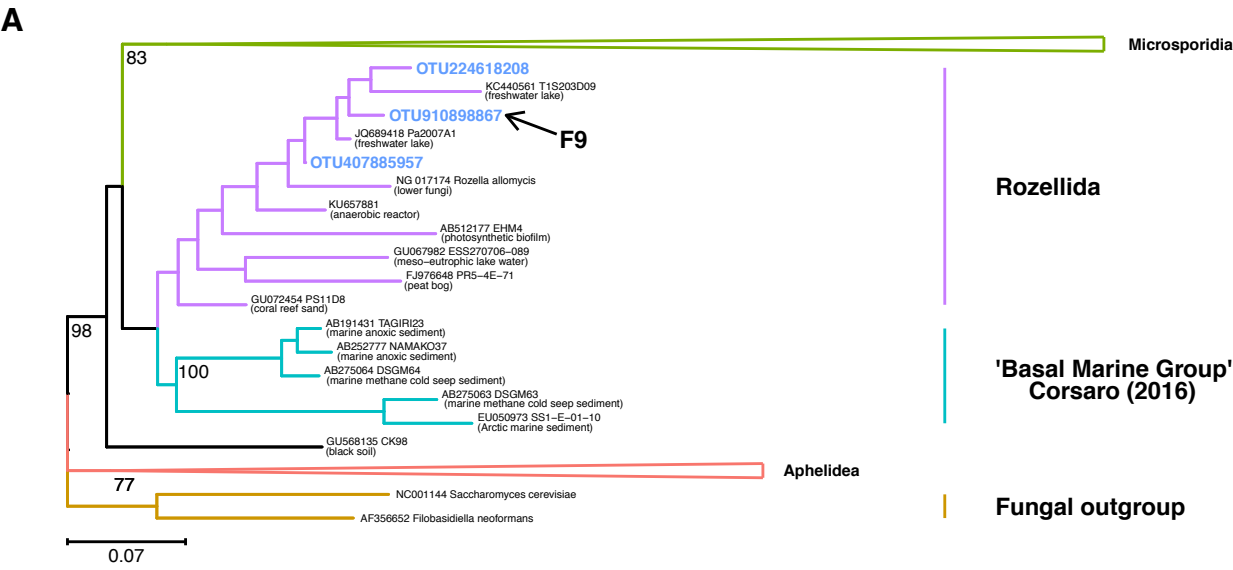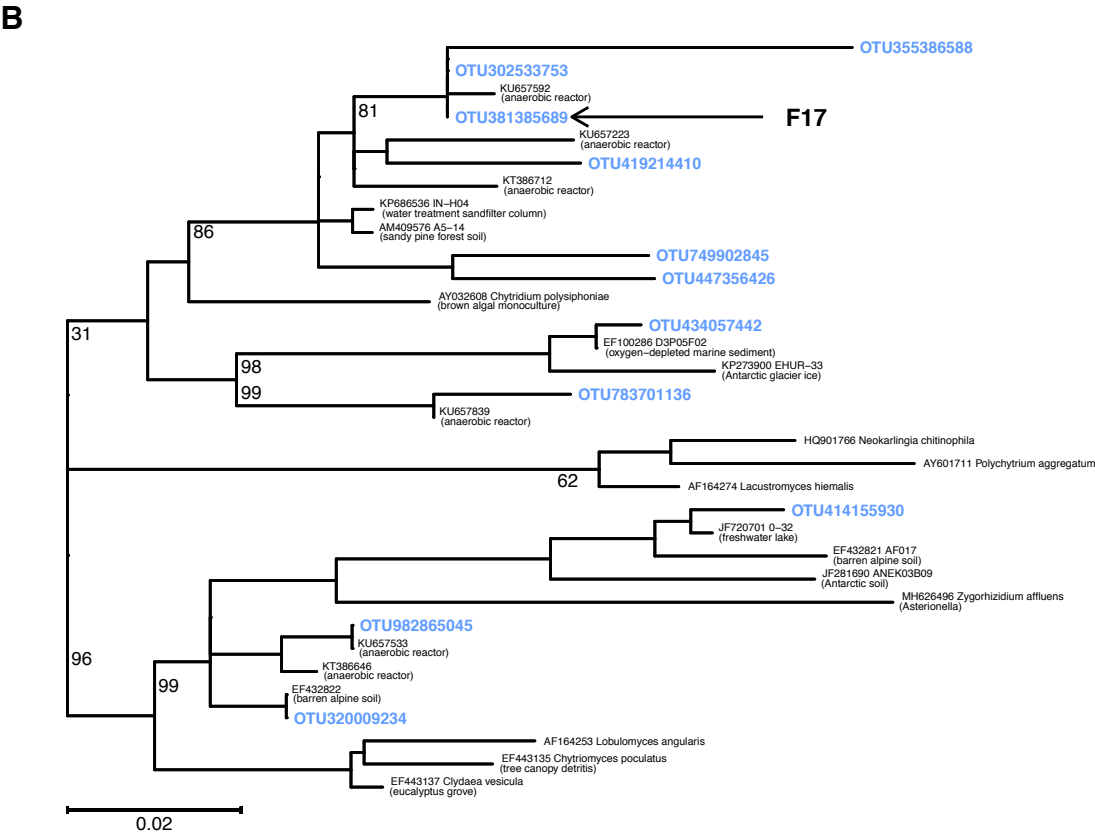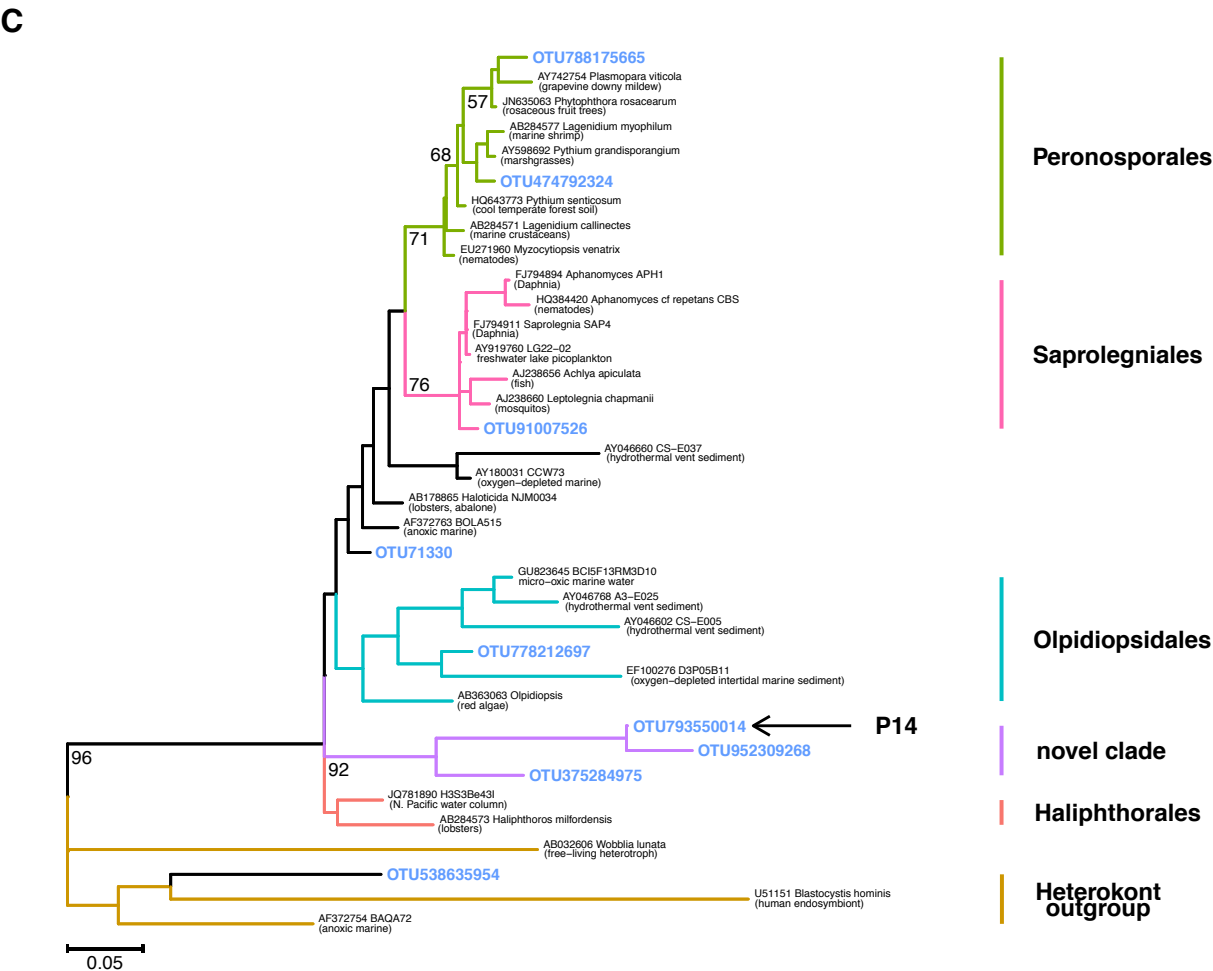

Fig. S3

Supplement: Supplementary file 9 — Additional file 8: Supplementary Figure 3. Phylogenetic tree of 18S rRNA sequences a) including the 18S sequence of the fungi (a) F9 and (b) F17 and the novel protist (c) P14. Other closely related less abundant OTUs found in this study are also included and highlighted in blue. The hosts the lineages are associated, or which environments they were sequenced from are in parenthesis. [file 40168_2020_972_MOESM9_ESM.pdf]

Fig. S4

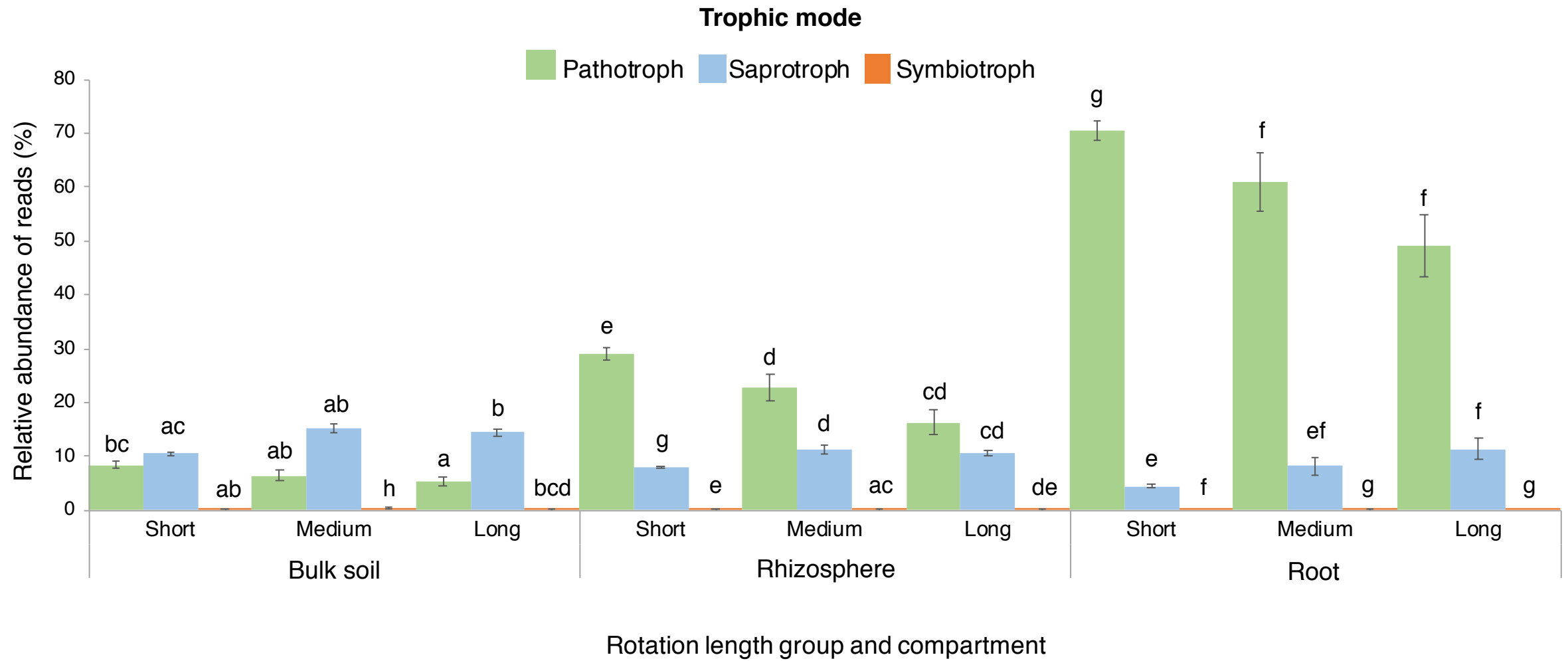

Supplement: Supplementary file 10 — Additional file 9: Supplementary Figure 4. Relative abundance of trophic modes based on FUNguild determinations. Error bars represent standard error of the mean. Different letters above the bars indicate significant differences at the P < 0.05 level between bars of the same trophic mode. Rotations lengths were binned into groups of short (1 in 2 years to 1 in 4 years), medium (1 in 5 years to 1 in 7 years) and long (1 in 8 years and longer). [file 40168_2020_972_MOESM10_ESM.pdf]

**Fig. S5**

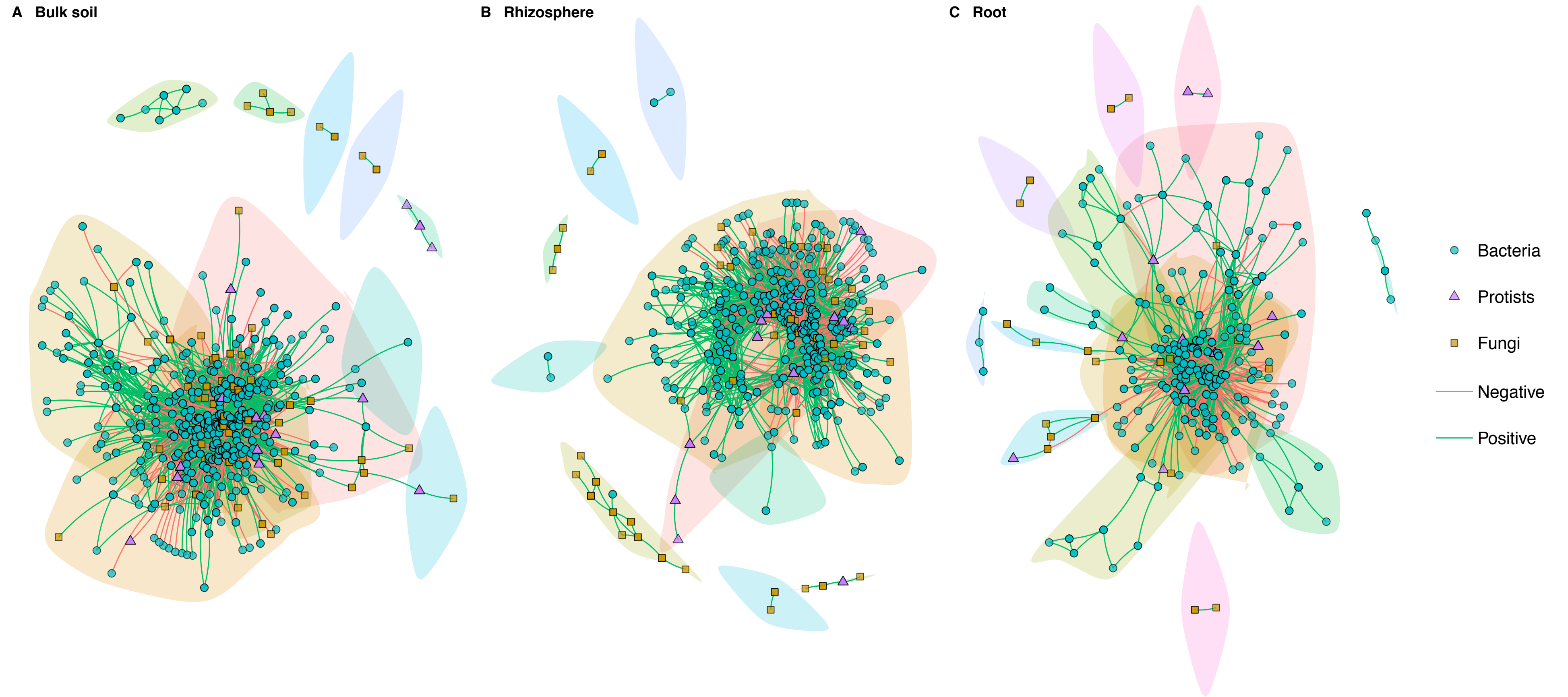

Supplement: Supplementary file 11 — Additional file 10: Supplementary Figure 5. Correlation networks generated using SparCC. Edges indicate correlation of >0.4 or <-0.4. (a) bulk soil, (b) rhizosphere soil, (c) root. [file 40168_2020_972_MOESM11_ESM.pdf]
